# Effects of exercise on bone mass and bone metabolism in adolescents: a systematic review and meta-analysis

**DOI:** 10.3389/fphys.2024.1512822

**Published:** 2024-12-24

**Authors:** Wenhua Zhang, Xiaoqiang Wang, Yurong Liu, Qiang He, Qixin Ding, Jingqi Mei, Xun Li

**Affiliations:** ^1^ School of Graduate Education, Shandong Sport University, Jinan, Shandong, China; ^2^ School of Sport and Health, Shandong Sport University, Jinan, Shandong, China; ^3^ Department of Basic Medicine, College of Medicine, Hunan Normal University, Changsha, Hunan, China; ^4^ School of Physical Education, Shandong University, Jinan, Shandong, China; ^5^ School of Rehabilitation, Henan University of Chinese Medicine, Zhengzhou, Henan, China; ^6^ Department of Nursing, College of Medicine, Hunan Normal University, Changsha, Hunan, China

**Keywords:** exercise, physical activity, adolescents, bone mineral content, bone mineral density, bone metabolism, meta-analysis, randomized controlled trial

## Abstract

**Objective:**

Meta-analysis was used to evaluate the effects of an exercise intervention on bone mineral content (BMC), bone mineral density (BMD), and bone metabolism in adolescents.

**Methods:**

A systematic search of PubMed, Embase, Cochrane Library, and Web of Science for RCTs on “exercise, adolescents, BMD, bone metabolism” up to 10 September 2024. Included RCTs focused on effects of exercise on BMC, BMD, and bone metabolism in 10–19 years old, with physical activity as exercise group and daily living/primal exercise as control group. Outcome metrics included lumbar spine, femoral neck, whole body BMC and BMD, bone alkaline phosphatase (BALP), procollagen type 1N-terminal propeptide (PINP), osteocalcin (OC), and type I collagen carboxy-terminal peptide (CTX). Exclusion criteria included duplicates, non-RCTs, non-adolescent studies, and non-compliance with indicators. Meta-analyses were performed using RevMan 5.4, and quality assessed by Cochrane’s tool. Effect sizes were estimated using standardized mean differences (*SMDs*) and 95% confidence intervals (*CIs*), and heterogeneity was assessed using the *I* ^*2*^ statistic to determine fixed or random effects models.

**Results:**

Fifteen articles with a total of 723 subjects were included. The meta-analysis showed that, compared to the control group, (i) exercise was effective in increasing adolescents’ overall BMC (*SMD* = 0.16, 95% *CI*: 0.06–0.27, *p* = 0.003) and BMD (*SMD* = 0.26, 95% *CI*: 0.13–0.40, *p* = 0.0001). (ii) Subgroup analyses showed that exercise significantly increased adolescents’ lumbar spine BMC (*SMD* = 0.17, 95% *CI*: 0.01–0.34, *p* = 0.04), femoral neck BMC (*SMD* = 0.23, 95% *CI*: 0.05–0.42, *p* = 0.01), lumbar spine BMD (*SMD* = 0.34, 95% *CI*: 0.12–0.56, *p* = 0.003) and femoral neck BMD (*SMD* = 0.31, 95% *CI*: 0.09–0.53, *p* = 0.007), whereas there was no statistically significant effect on whole body BMC and BMD (*p* > 0.05). (iii) Exercise increased BALP, and decreased PINP, OC and CTX in adolescents. but none of the differences between the exercise groups and the control group were significant (*p* > 0.05).

**Conclusion:**

Exercise is effective in improving overall BMC and BMD in adolescents and elevating BMC and BMD of the lumbar spine and femoral neck. Due to the limitation of the number and quality of the included studies, the above conclusions are yet to be validated by more high-quality empirical studies.

**Systematic Review Registration:**

https://www.crd.york.ac.uk/, identifier CRD42024593399

## 1 Introduction

Bone mineral density (BMD), as a key measure of bone mineral content (BMC) per unit volume, can accurately and sensitively reflect an individual’s bone health, and is an indispensable ruler for assessing bone growth and development (Chevalley and Rizzoli, 2022). During early childhood and adolescence, a period of rapid growth, bone mass can be assessed by measuring BMC and BMD (Wolff et al., 1999). The accumulation of bone mass is particularly critical, especially during puberty, when the rate of bone mass accumulation accelerates significantly, reaching approximately 50% of peak bone mass during this period. Toward the end of puberty, the accumulation of bone mass is nearly complete, usually reaching about 90% of peak bone mass (Kalkwarf et al., 2022). Therefore, adequate acquisition of peak bone mass during adolescence is crucial and decisive in preventing the risk of osteoporotic fractures in adulthood. In addition, markers of bone metabolism in adolescence are important because they reflect the dynamic process of bone growth and remodeling, help assess BMD and fracture risk, and play a key role in preventing osteoporosis and promoting healthy bone development.

The underlying mechanism by which exercise can increase BMD and BMC involves multiple physiological pathways: first, mechanical loads act on the skeleton through muscle forces and ground reaction forces to stimulate osteoblasts, thereby promoting bone formation (Troib et al., 2016; Prawiradilaga et al., 2020; Lara-Castillo et al., 2023); Secondly, exercise also promotes the secretion of certain hormones, such as growth hormone and sex hormones, which are essential for bone growth and maintenance of bone mass (Troib et al., 2016; Xiao et al., 2016; Hughes et al., 2023); Furthermore, exercise promotes osteoblast differentiation and increases bone mass by up-regulating Wnt3a/β-catenin and other related signaling pathways (Chen et al., 2021); Finally, regular exercise helps to maintain and increase muscle strength, and the force generated by muscle contraction is applied to the bones, which further stimulates the increase in bone mass and reduces bone loss (Kim et al., 2012; Xiao et al., 2016). In addition, exercise can also improve bone metabolism through certain underlying mechanism. Some studies have reported that exercise can induce muscle and fat to secrete various factors, such as irisin and leptin (Alizadeh Pahlavani, 2022), and can also affect the secretory function of bone tissue to regulate bone metabolism (Dalle Carbonare et al., 2023). Exercise also affects bone metabolism by promoting mechanical loading of muscles, activating brown adipose tissue, and regulating autophagy in osteoblasts (Di Maio et al., 2022; Zhang L. et al., 2022). Together, these combined effects promote an increase in BMC, BMD and bone metabolism, which enhances bone strength and stability.

This review focuses on how exercise affects bone health in the adolescent population, specifically as measured by the key metrics of BMC and BMD, which reflect the static quality of the skeleton. Since BMC and BMD do not change significantly over a 12-month period, frequent measurements are not recommended. In order to look more dynamically at the effects of exercise on bone reconstruction, the study used bone metabolism markers, which can show significant changes over a period of 3–6 months. In addition, as bone metabolic activity increases with age, the potential for clinical use of these markers in assessing fracture risk warrants attention (Greenblatt et al., 2017).

Appropriate exercise plays a good role in promoting bone growth and development (Otsuka et al., 2021). Exercise is closely related to bone mass and bone metabolism, and within a certain intensity range, physical activity has a significant improvement effect on both bone mass and bone metabolism in adolescents, but the effects of exercise on BMC and BMD in different parts of adolescents as well as the effects of exercise on bone metabolism markers in adolescents remain to be clarified. Scientific and rational exercise interventions using more effective exercises at the adolescent stage play an important role in increasing their peak bone mass, improving bone metabolism, and thus preventing senile osteoporosis (Berro et al., 2024). A large number of studies have confirmed that aerobic exercise (Snow-Harter et al., 1992), resistance exercise (Kox et al., 2018), impact exercise (Lima et al., 2001; Vlachopoulos et al., 2015; Kurgan et al., 2022), and a combination of various types of exercise (Campos et al., 2014) during adolescence can Significantly promoted improvements in BMC, BMD and bone metabolism indices. Therefore, the article builds on previous studies by incorporating more exercises (moderate-intensity continuous training, whole-body vibration training, core stability training, high-intensity interval training, jumping training, AquaPlyo training, etc.) related to BMC, BMD and bone metabolism indices in adolescents, where peak bone mass gained from sports exercise during adolescence reduces the risk of fracture in old age by 50% (Tveit et al., 2015; Correa-Rodríguez et al., 2016). Therefore, increasing peak bone mass in adolescence is an effective means of preventing osteoporosis. In this paper, we use the method of meta-analysis to provide theoretical basis for adolescents to exercise scientifically to prevent the occurrence of osteoporosis in old age by combing the randomized controlled trial (RCT) on the effect of exercise intervention on BMC, BMD and bone metabolism markers in adolescents.

## 2 Materials and methods

### 2.1 Protocol and registration

The present comprehensive systematic review and meta-analysis adheres to the guidelines outlined in the Preferred Reporting Items for Systematic Reviews and Meta-Analyses (PRISMA) Statement (Page et al., 2021). The registration of this review took place in the International Prospective Register of Systematic Reviews (PROSPERO, No. CRD42024593399), adhering to the guidelines for preferred reporting items in systematic reviews and meta-analyses.

### 2.2 Search strategy

Retrieved September 2024 by first author. PubMed, Embase, Cochrane Library, Web of Science databases were searched. RCTs on the effects of exercise on BMC and BMD in adolescents were searched for each database. Search terms included “Exercise, Physical Exercise, Aerobic Exercise, Resistance Training, Impact Exercises, Adolescent, Bone Mineral Density, Randomized Controlled Trial, Bone Mineral Content, bone metabolism”. Articles were searched by using the following search criteria: (exercise [MeSH] OR Physical Exercise OR Physical Activity OR Aerobic Exercise OR Isometric Exercise OR Acute Exercise OR Exercise Training) AND (Bone Mineral Density [MeSH] OR Bone Mineral Content) AND (Bone Metabolism [MeSH]) AND (Adolescents [MeSH] OR Youth OR Teenager OR Teen) AND (Randomized Controlled Trial [MeSH] OR Controlled Clinical Trial OR Clinical Study).

### 2.3 Inclusion and exclusion criteria for the studies

The inclusion criteria were (i) Type of study: Published RCTs of the effects of exercise on BMC, BMD and bone metabolism in adolescents from the time of construction to 10 September 2024, for each database. (ii) Subjects: adolescents of any gender who met the World Health Organization’s age definition (10–19 years old) (Organization, 2006). (iii) Intervention: any form of physical activity (which is a cultural activity in which participants are physically active through significant physical movement for the purpose of strengthening their physical fitness and improving their health), with physical activity as the main activity in the intervention group. Control measures: The control group only performed daily life or original physical exercise and did not receive additional exercise intervention. (iv) Outcome indicators: lumbar spine, femoral neck, whole body BMC and BMD, bone alkaline phosphatase (BALP), procollagen type 1N-terminal propeptide (PINP), osteocalcin (OC), type I collagen carboxy-terminal peptide (CTX). The exclusion criteria were (i) duplication of published literature; (ii) non-RCT; (iii) non-adolescents in the study; and (iv) non-compliance of outcome indicators.

### 2.4 Literature screening and data extraction

The retrieved literature was imported into Endnote 20 software and duplicates were removed from it. Subsequently, two researchers screened the literature and extracted information based on the established inclusion and exclusion criteria. Where disagreements were encountered, they were resolved through discussion with the 3rd researcher. Characteristics of the included studies included author, year of publication, country and region, intervention, sample size, age, exercise status during the trial in the control group, and outcome indicators (Tables 1, 2). Data were extracted as pre- and post-intervention means and standard deviations (*SD*). Dependent variables included BMC or BMD reported in g or g/cm^2^. The lumbar spine, femoral neck, and whole-body BMC and BMD covered in this article were measured using dual-energy X-ray absorptiometry (DXA).

**TABLE 1:** Study characteristics.

Athor	Year	Country and area	Sample population (n)		Age (Years)	
			EG	CG	EG	CG
Eliakim et al. (1997)	1997	United States	20	18	16 ± 0.7	
Nichols et al. (2001)	2001	United States	5	11	17.4 ± 0.4	16.8 ± 0.3
Arnett and Lutz (2002)	2002	United States	13	12	15.2 ± 0.6	15.0 ± 0.9
			12		14.8 ± 0.7	
Kontulainen et al. (2002)	2002	Filand	50	49	14.5 ± 1.5	13.8 ± 1.6
Stear et al. (2003)	2003	United States	38	28	17.3 ± 0.4	17.4 ± 0.3
Ferry et al. (2014)	2014	United States	20	22 (21)	16 ± 1.8	16.9 ± 1.5
Nogueira et al. (2014)	2014	Australia	12	6	11.3 ± 0.6	11.4 ± 0.6
Zribi et al. (2014)	2014	Tunisia	25	26	12.3 ± 0.5	12.4 ± 0.6
Martin et al. (2017)	2017	United States	20	21	16.8 ± 2.4	16.8 ± 2.3
Vlachopoulos et al. (2018)	2018	United Kingdom	SWI: 19 FOO: 15 CYC: 14	SWI: 18 FOO: 15 CYC: 12	SWI: 15.3 ± 0.9 FOO: 14.6 ± 1.0 CYC: 14.9 ± 1.1	SWI: 15.4 ± 1.1 FOO: 14.5 ± 0.8 CYC: 14.9 ± 0.9
Marin-Puyalto et al. (2020)	2020	Spain	34	28	14.4 ± 1.9	14.1 ± 1.8
Elnaggar et al. (2021)	2021	Egypt	17	16	12.11 ± 1.65	11.31 ± 1.35
Lau et al. (2021)	2021	Hong Kong, China	14	16	12.8 ± 0.9	13.2 ± 1.1
Julian et al. (2022)	2022	France	19	11	13.0 ± 1.1	13.2 ± 1.0
			19		13.0 ± 0.8	
Elnaggar and Elfakharany (2023)	2023	Egypt	24	24	14.25 ± 2.01	13.75 ± 1.48

Table Note: EG, exercise group; CG, control group; SWI, swimmers; FOO, footballers; CYC, cyclists.

**TABLE 2:** Characteristics of the study intervention.

Author	Interventions	Exercise status of the control group during the trial	Intervention cycle	Duration of one intervention	Frequency of intervention	Endpoint indicators (BMC, BMD) units (g, g/cm^2^)
Eliakim et al. (1997)	Endurance-based training, including running, aerobics, competitive sports (e.g., basketball) and occasional weight lifting	Former sports	5 weeks	2 h	5 times per week	⑦⑨⑩
Nichols et al. (2001)	Resistance exercise	Sedentary lifestyle	8 months	30–40 min	3 times per week	①②③④⑤⑥
Arnett and Lutz (2002)	Rope skipping (HV) and rope skipping (LV)	Regular physical exercise	4 months	10 min and 5 min respectively	4 times per week	①②
Kontulainen et al. (2002)	Aerobics and jumps	Physical inactivity	9 months	50 min	2 times per week	①②
Stear et al. (2003)	Aerobic, weight-bearing, impact exercise	Physical inactivity	15.5 months	45 min	3 times per week	①③
Ferry et al. (2014)	Moderate intensity exercise to vigorous exercise with gravitational impact	Physical inactivity	12 months	1 h	2 times per week	①②④⑤
Nogueira et al. (2014)	Jumping and capoeira	Regular physical exercise	9 months	10 min	3 times per week	①②③④⑤⑥
Zribi et al. (2014)	Jumping jacks	Former sports	9 weeks	NR	2 times per week	①②③④⑤⑥
Martin et al. (2017)	High-impact, low-frequency movements, specifically vertical jumps	Routine care	Time 2: 4–6 days Time 3: 7–9 days	5 min	2 times per day	⑦⑨
(Vlachopoulos et al., 2018)	Plyometric exercise, specifically countermovement jumps	Continued with their motor training routine without additional jump training interventions	9 months	10 min	3 times per week	⑦⑩
Marin-Puyalto et al. (2020)	Whole body vibration training	Former sports	6 months	NR	3 times per week	①②③④⑤⑥⑧⑨⑩
Elnaggar et al. (2021)	Core stability training	Regular physical exercise	3 months	45 min	3 times per week	④⑤
Lau et al. (2021)	High intensity interval training	Regular physical exercise	6 months	NR	5 times per week	③⑥
Julian et al. (2022)	High intensity interval training and moderate intensity continuous training	Regular physical exercise	16 weeks	15 and 45 min, respectively	2 times per week	①③④⑤⑥
Elnaggar and Elfakharany (2023)	AquaPlyo training	Standardized training	12 weeks	45 min	2 times per week	④⑤

Table Note: BMC, bone mineral content; BMD, bone mineral density; HV, high volume; LV, low volum; NR, not reported; ①, lumbar spine BMC; ②, femoral neck BMC; ③, whole body BMC; ④, lumbar spine BMD; ⑤, femoral neck BMD; ⑥, whole body BMD; ⑦, bone alkaline phosphatase (BALP); ⑧, procollagen type 1N-terminal propeptide (PINP); ⑨, osteocalcin (OC); ⑩, type I collagen carboxy-terminal peptide (CTX).

### 2.5 Quality assessment

For RCTs and controlled clinical trials, the risk of bias was provided by Review Manager 5.4 (The Cochrane Collaboration, Oxford, England) (Higgins et al., 2011). Two researchers each assessed the risk of bias in the selected literature using the Cochrane Risk of Bias Assessment Tool. The assessment covered the generation of randomized sequences, allocation concealment, blinding of participants and researchers, blinding of evaluators, completeness of endpoints, selective reporting of results, and other potential sources of bias. Each element was assessed as high risk of bias, low risk of bias, or unknown risk of bias. Disagreements during the assessment process were resolved by discussion.

### 2.6 Data analysis

Meta-analysis of the included outcome indicators was performed using Review Manager 5.4 software package. The outcome indicators of the literature included in the data of this study were continuous variables, and the standardized mean difference (*SMD*) and 95% confidence interval (95% *CI*) were selected as the effect scales for the combined effect sizes. Statistical inferences were made by heterogeneity test and statistical combined effect sizes. Heterogeneity was tested by the *p*-value of *Chi* ^*2*^ and *I* ^*2*^. In the heterogeneity test, *p* > 0.10 means that the heterogeneity of the literature included in this study is negligible, and *p* ≤ 0.10 means that the heterogeneity of the literature included in this study exists. The judgment of heterogeneity is dominated by the value of *I* ^*2*^ when *p* ≤ 0.10. with 0 ≤ *I* ^*2*^ ≤ 25% to ignore heterogeneity, 25% < *I* ^*2*^ ≤ 50% to indicate mild heterogeneity in the included literature, 50% < *I* ^*2*^ ≤ 75% to indicate moderate heterogeneity in the included studies, and *I* ^*2*^ > 75% to indicate a high degree of heterogeneity among the included studies. Literature with moderate to high heterogeneity was analyzed using a random effects model, and literature with mild or negligible heterogeneity was analyzed using a fixed effects model. Data presentation was achieved using forest plots, and publication bias was identified and analyzed using funnel plots.

## 3 Results

### 3.1 Search result

**FIGURE 1:**
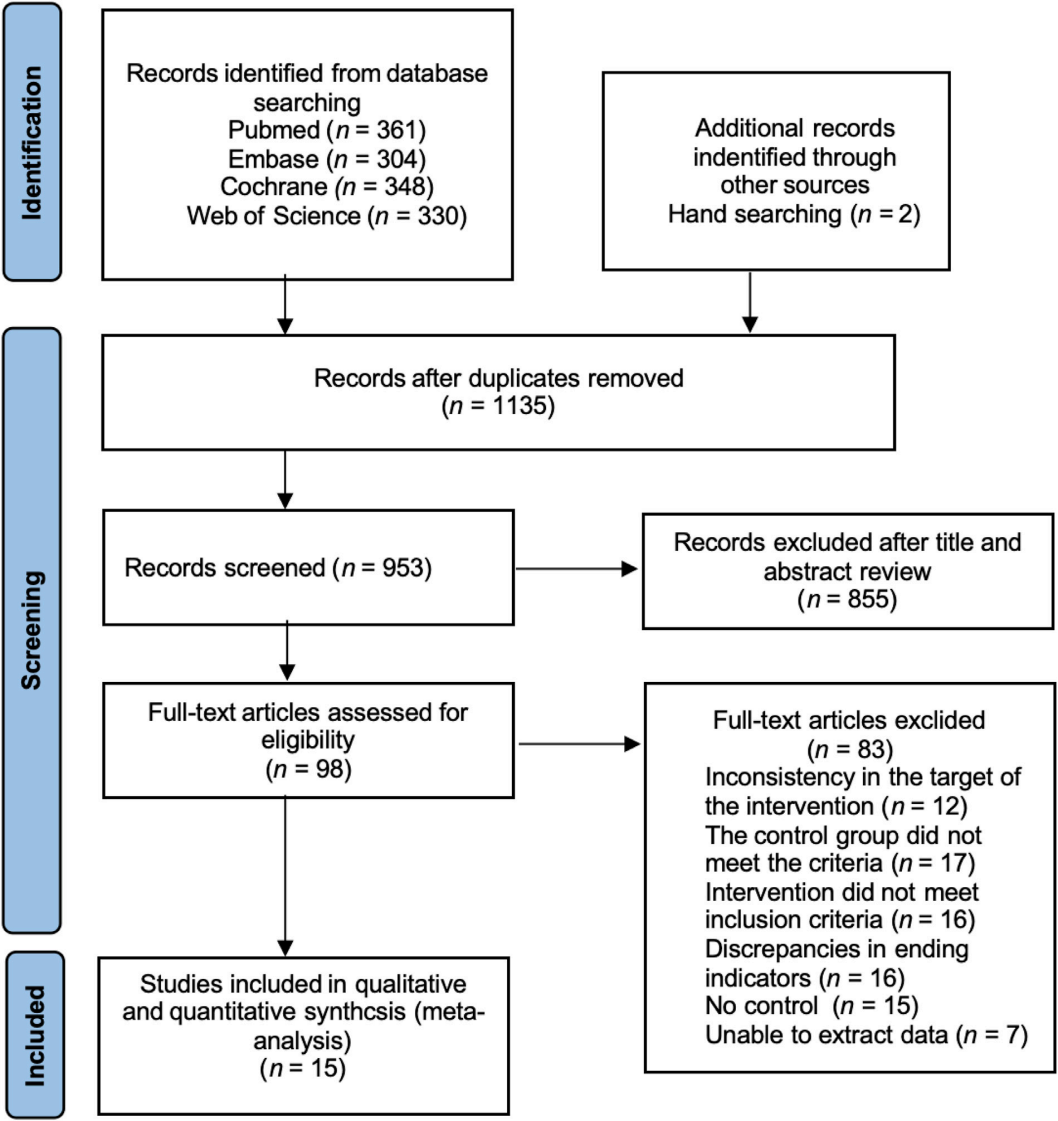
PRISMA study flow diagram.

The initial screening identified 1,345 articles in the relevant literature, including 361 articles in PubMed database, 304 articles in Cochrane Library database, 348 articles in Embase database, 330 articles in Web of Science. The EndNote20 software de-emphasized 182 articles, 855 articles were excluded after reading the titles and abstracts, 6 articles with low relevance were excluded, and the remaining 98 articles were read through the full text to assess whether they were included or not. Among them, 12 interventions did not match the target group, 17 controls did not meet the criteria, 16 interventions did not meet the inclusion criteria, 16 outcome indicators did not match, 15 had no control, 7 could not extract data, and finally the remaining 15 articles were included in meta-analysis (Eliakim et al., 1997; Nichols et al., 2001; Arnett and Lutz, 2002; Kontulainen et al., 2002; Stear et al., 2003; Ferry et al., 2014; Nogueira et al., 2014; Zribi et al., 2014; Martin et al., 2017; Vlachopoulos et al., 2018; Marin-Puyalto et al., 2020; Elnaggar et al., 2021; Lau et al., 2021; Julian et al., 2022; Elnaggar and Elfakharany, 2023) (Figure 1).

### 3.2 Characteristics of included studies

Fifteen articles were included in the study, published between 1997 and 2023, with a total of 723 subjects (390 in the experimental group and 333 in the control group), aged 10–19 years (Table 1). Of these, 6 were from the United States (Eliakim et al., 1997; Nichols et al., 2001; Arnett and Lutz, 2002; Stear et al., 2003; Ferry et al., 2014; Martin et al., 2017), 2 from Egypt (Elnaggar et al., 2021; Elnaggar and Elfakharany, 2023), 1 from United Kingdom (Vlachopoulos et al., 2018), 1 from France (Julian et al., 2022), 1 from Finland (Kontulainen et al., 2002), 1 from Australia (Nogueira et al., 2014), 1 from Tunisia (Zribi et al., 2014), 1 from Spain (Marin-Puyalto et al., 2020), and 1 from Hong Kong, China (Lau et al., 2021) (Table 1). The intervention group included 2 resistance exercises (Nichols et al., 2001), 4 combination exercises (Eliakim et al., 1997; Kontulainen et al., 2002; Stear et al., 2003; Ferry et al., 2014; Nogueira et al., 2014), 1 whole body vibration training (Marin-Puyalto et al., 2020), 2 high-intensity interval training exercises (Lau et al., 2021; Julian et al., 2022), 3 jumping exercise (Zribi et al., 2014; Martin et al., 2017; Vlachopoulos et al., 2018), 1 core stability training (Elnaggar et al., 2021), 1 moderate-intensity continuous training (Julian et al., 2022), 1 rope skipping exercise (Arnett and Lutz, 2002), 1 AquaPlyo training (Elnaggar and Elfakharany, 2023) (Table 2). The intervention period was 9 weeks–15.5 months, with an exercise frequency of 2-5 sessions/week and an exercise duration of 5–60 min/session (Table 2).

### 3.3 Risk of bias

**FIGURE 2:**
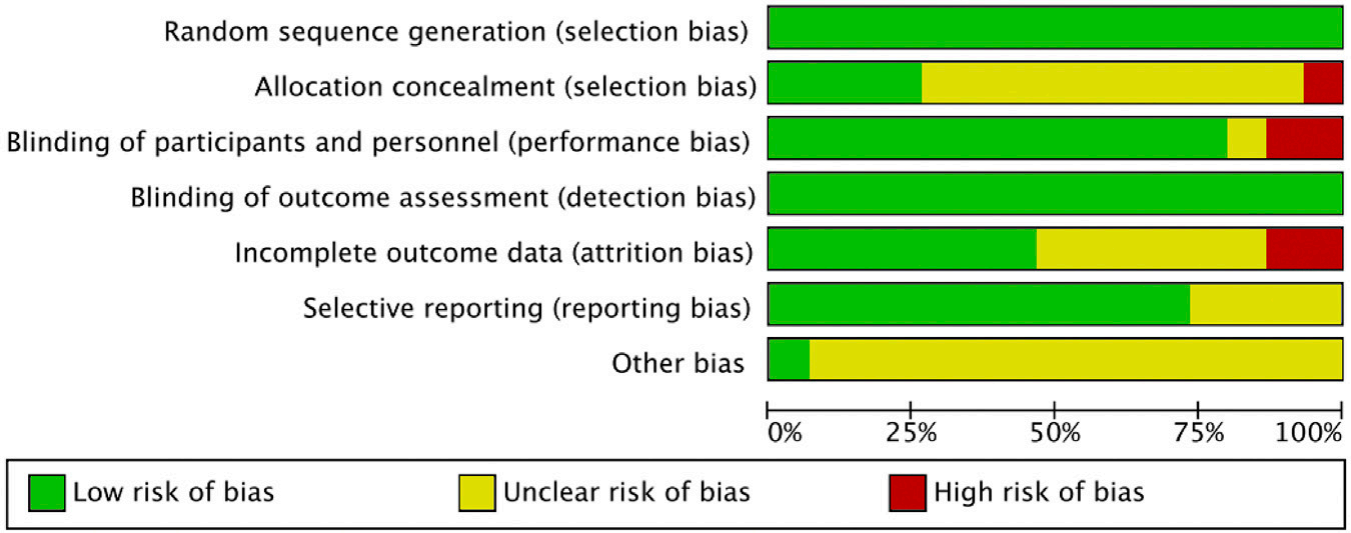
Risk of bias of the included studies.

**FIGURE 3:**
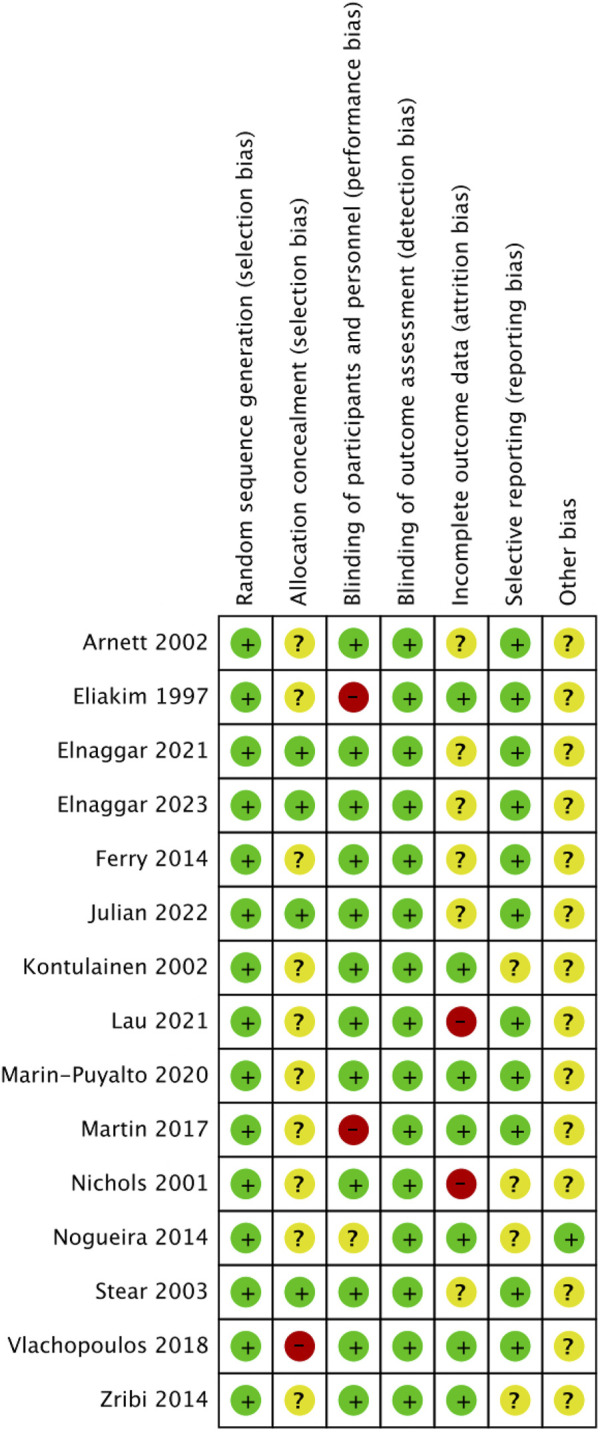
Risk of bias summary of the included studies. (√), Low risk; (×), High risk; (?), unclear or inadequately described.

The included literature was all RCTs, and the overall quality of the literature was high. The risk of bias is summarized in Figures 2, 3. The quality of 15 studies (Eliakim et al., 1997; Nichols et al., 2001; Arnett and Lutz, 2002; Kontulainen et al., 2002; Stear et al., 2003; Ferry et al., 2014; Nogueira et al., 2014; Zribi et al., 2014; Martin et al., 2017; Vlachopoulos et al., 2018; Marin-Puyalto et al., 2020; Elnaggar et al., 2021; Lau et al., 2021; Julian et al., 2022; Elnaggar and Elfakharany, 2023) was assessed using the Cochrane Systematic Evaluation Tool version 5.1.0, and the results were as follows: random sequence generation (low, 15), allocation concealment (low, 4; uncertain, 10; high, 1), blinding of participants and personnel (low, 12; uncertain, 1; high, 2), blinding outcome assessment (low, 15), incomplete outcome data (low, 7; uncertain, 6; high, 2), selective reporting (low, 11; uncertain, 4), and other biases (low, 1; uncertain, 14).

### 3.4 Meta-analysis results

#### 3.4.1 Effect of exercise on bone mineral content indicators in adolescents

**FIGURE 4:**
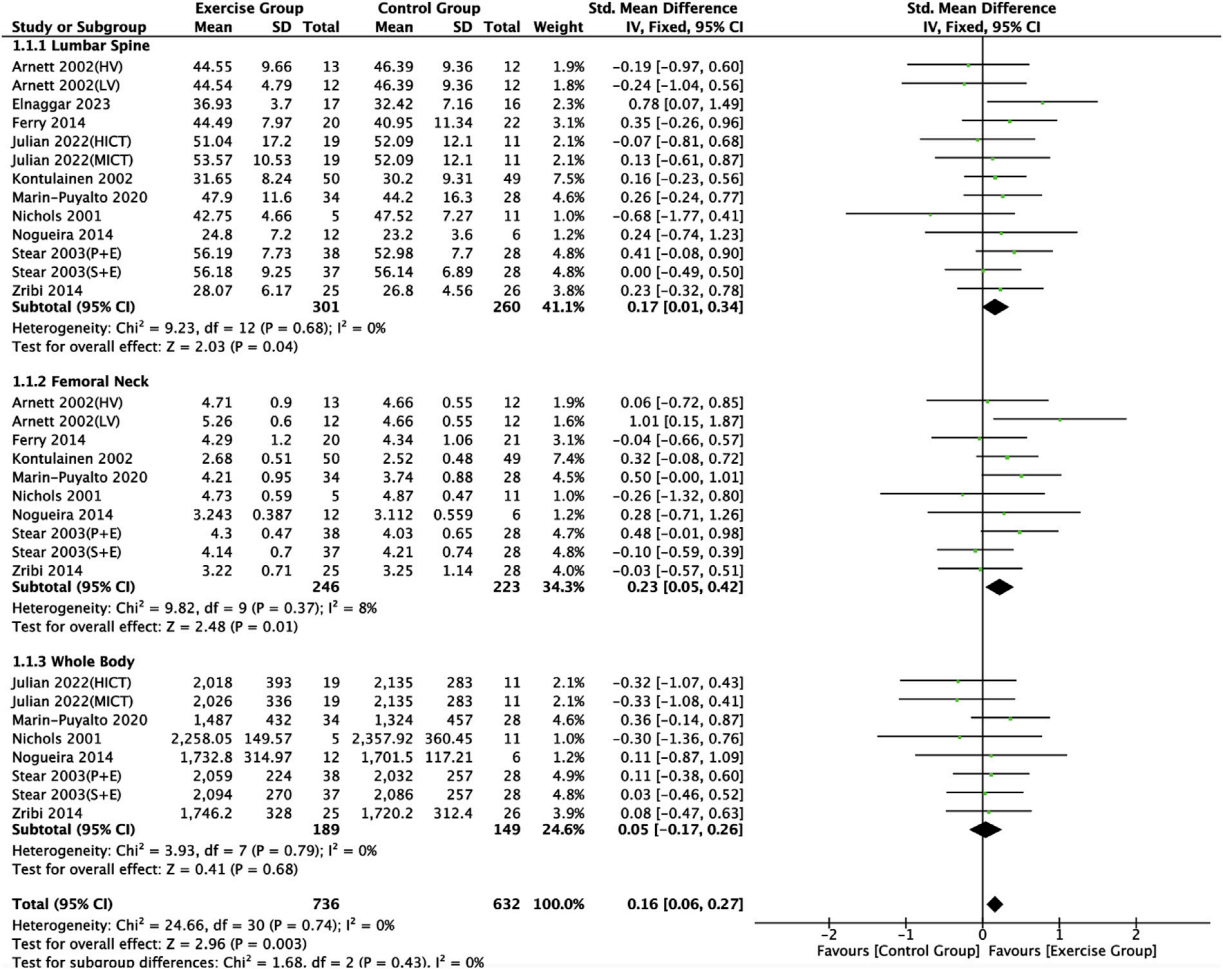
Meta analysis forest plot of the impact of exercise on bone mineral content indicators. HV, high volume; LV, low volume; P + E, placebo + exercise; S + E, calcium supplement + exercise; HIIT, high intensity interval training; MICT, moderate intensity continuous training.

**FIGURE 5:**
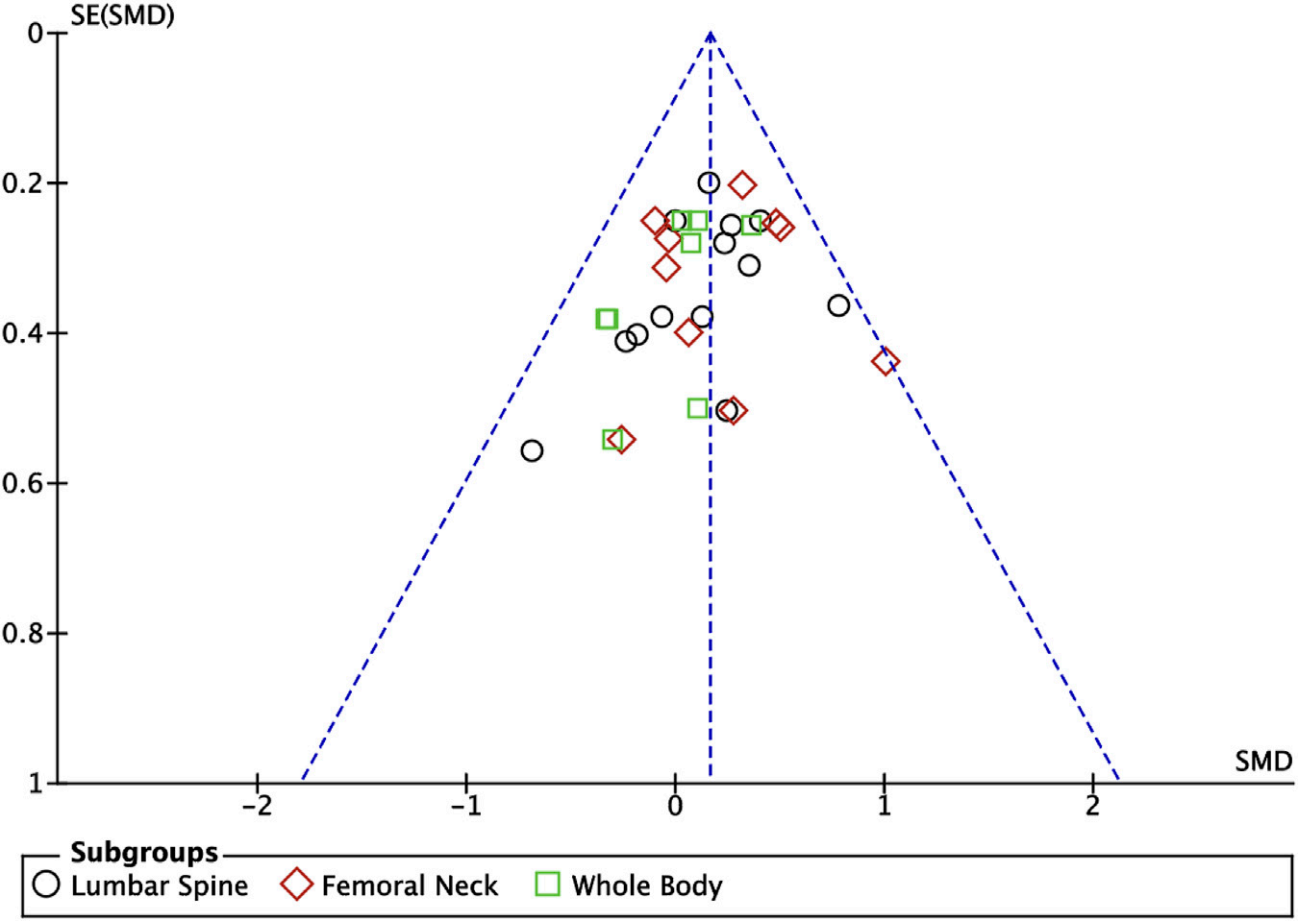
Meta analysis funnel plot of the effect of exercise on bone mineral content indicators.

Ten articles (Nichols et al., 2001; Arnett and Lutz, 2002; Kontulainen et al., 2002; Stear et al., 2003; Ferry et al., 2014; Nogueira et al., 2014; Zribi et al., 2014; Marin-Puyalto et al., 2020; Lau et al., 2021; Julian et al., 2022) included in this study described the effects of exercise on BMC in adolescents, of which nine articles (Nichols et al., 2001; Arnett and Lutz, 2002; Kontulainen et al., 2002; Stear et al., 2003; Ferry et al., 2014; Nogueira et al., 2014; Zribi et al., 2014; Marin-Puyalto et al., 2020; Julian et al., 2022) described the effects of exercise on lumbar spine BMC, seven articles (Nichols et al., 2001; Arnett and Lutz, 2002; Kontulainen et al., 2002; Ferry et al., 2014; Nogueira et al., 2014; Zribi et al., 2014; Marin-Puyalto et al., 2020) described the effects of exercise on femoral neck BMC, and seven articles (Nichols et al., 2001; Stear et al., 2003; Nogueira et al., 2014; Zribi et al., 2014; Marin-Puyalto et al., 2020; Lau et al., 2021; Julian et al., 2022) describing the effects of exercise on whole body BMC (Figure 4). Negligible heterogeneity between the results of the included studies was observed in the exercise intervention group compared to the control group (*I* ^*2*^ = 0%, *p* = 0.74), so the analysis was performed using a fixed-effects model, which showed that a significant increase in BMC levels occurred in the exercise intervention group compared to the control group (*SMD* = 0.16, 95% *CI*: 0.06–0.27, *p* = 0.003) (Figure 4). Subgroup analysis showed that there were significant differences in lumbar spine BMC (*SMD* = 0.17, 95% *CI*: 0.01–0.34, *p* = 0.04) and femoral neck BMC (*SMD* = 0.23, 95% *CI*: 0.05–0.42, *p* = 0.01) in the intervention group of adolescents compared to the control group after the exercise intervention (*p* < 0.05). Exercise tended to promote whole body BMC(*SMD* = 0.05, 95% *CI*: -0.17–0.26, *p* = 0.68) in adolescents, but the difference between the exercise intervention group and the control group was not significant (Figure 4). The funnel plots were largely symmetrical, indicating that there was no significant publication bias (Figure 5).

#### 3.4.2 Effect of exercise on bone mineral density indices in adolescents

**FIGURE 6:**
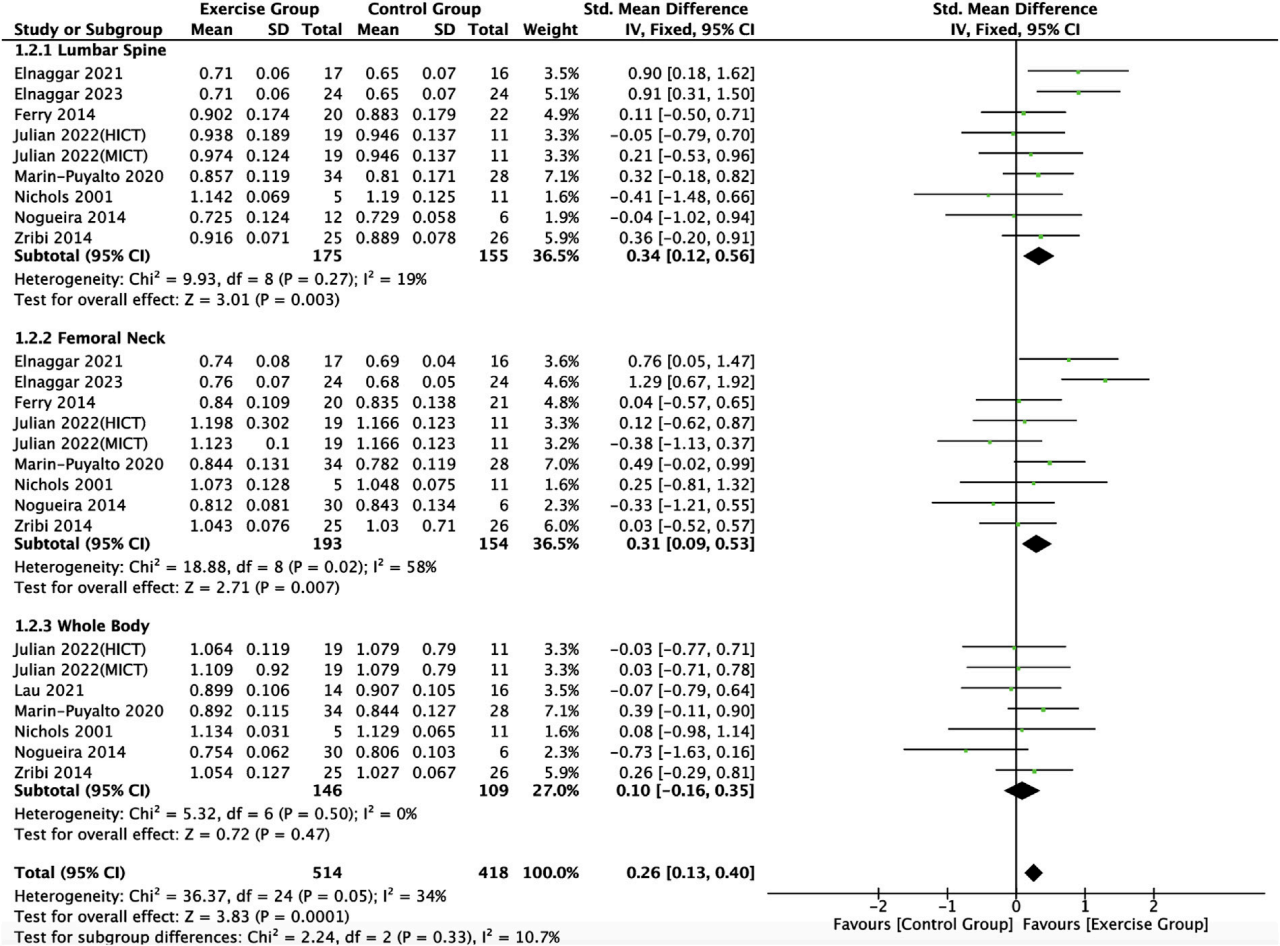
Meta analysis forest plot of the impact of exercise on bone mineral density indicators. HIIT, high intensity interval training; MICT, moderate intensity continuous training.

**FIGURE 7:**
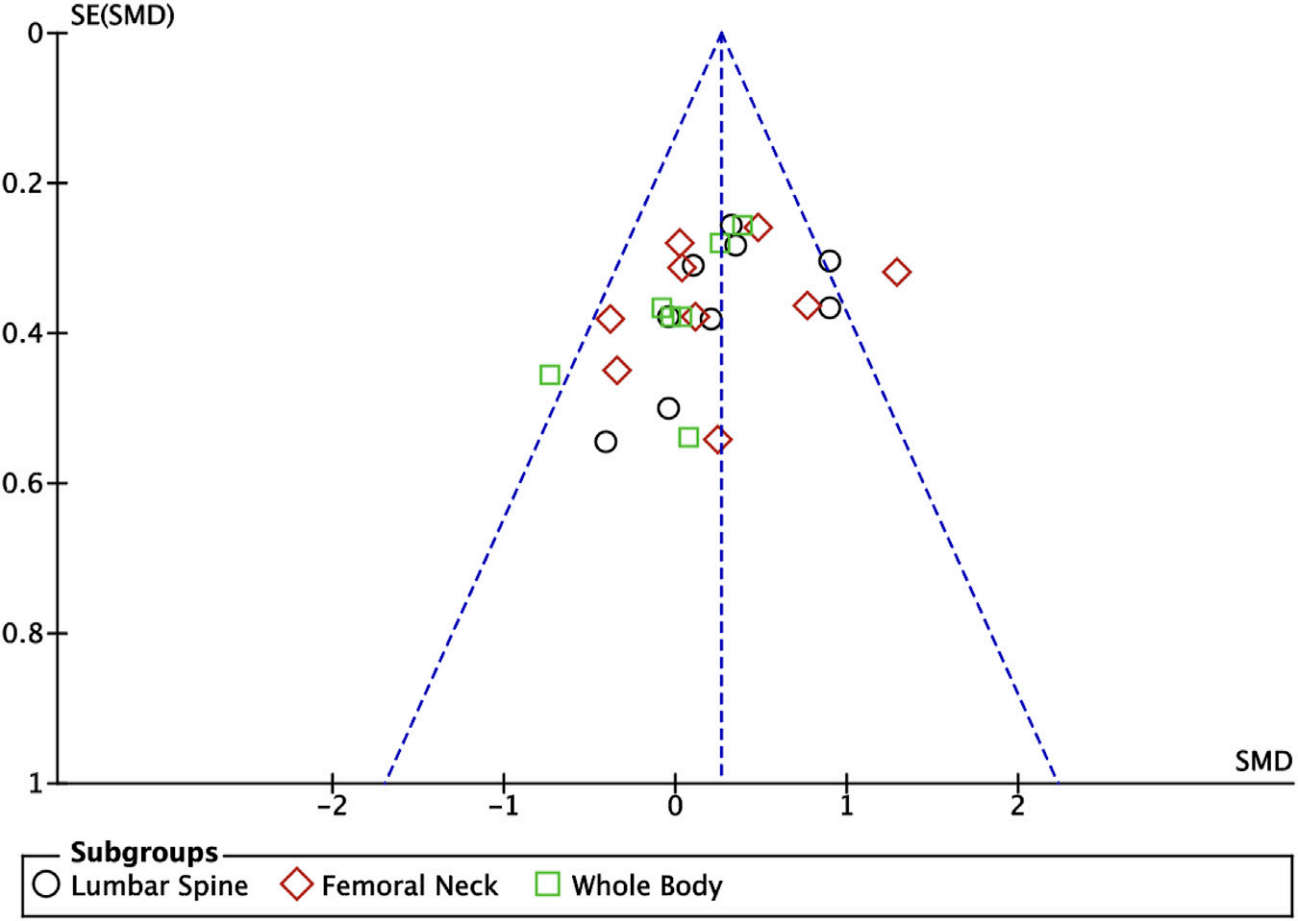
Meta analysis funnel plot of the impact of exercise on bone mineral density indicators.

Nine articles included in this study (Nichols et al., 2001; Ferry et al., 2014; Nogueira et al., 2014; Zribi et al., 2014; Marin-Puyalto et al., 2020; Elnaggar et al., 2021; Lau et al., 2021; Julian et al., 2022; Elnaggar and Elfakharany, 2023) described the effects of exercise on BMD in adolescents, eight of which (Nichols et al., 2001; Zribi et al., 2014; Marin-Puyalto et al., 2020; Elnaggar et al., 2021; Lau et al., 2021; Julian et al., 2022; Elnaggar and Elfakharany, 2023) described the effects of exercise on lumbar spine BMC, eight of which (Nichols et al., 2001; Ferry et al., 2014; Nogueira et al., 2014; Zribi et al., 2014; Marin-Puyalto et al., 2020; Elnaggar et al., 2021; Julian et al., 2022; Elnaggar and Elfakharany, 2023) described the effects of exercise on femoral neck BMD, and six of which (Nichols et al., 2001; Nogueira et al., 2014; Zribi et al., 2014; Marin-Puyalto et al., 2020; Lau et al., 2021; Julian et al., 2022) described the effect of exercise on whole-body BMD (Figure 6). Meta-analysis of forest plots showed mild heterogeneity between the results of the included studies in the exercise intervention group compared to the control group (*I* ^*2*^ = 34%, *p* = 0.05), so the analysis was performed using a fixed-effects model, and the results showed that there was a significant increase in the level of BMD in the exercise intervention group compared to the control group (*SMD* = 0.26, 95% *CI*: 0.13–0.40, *p* = 0.0001) (Figure 6). Subgroup analysis showed that there were significant differences in lumbar spine BMD (*SMD* = 0.34, 95% *CI*: 0.12–0.56, *p* = 0.003) and femoral neck BMD (*SMD* = 0.31, 95% *CI*: 0.09–0.53, *p* = 0.007) in the intervention group of adolescents compared to the control group after the exercise intervention (*p* < 0.05). Exercise tended to promote whole body BMD(*SMD* = 0.10, 95% *CI*: -0.16–0.35, *p* = 0.47) in adolescents, but the difference between the exercise intervention group and the control group was not significant (Figure 6). The funnel plot was essentially symmetrical, indicating that there was no significant publication bias (Figure 7).

#### 3.4.3 Effect of exercise on BALP in adolescents

**FIGURE 8:**
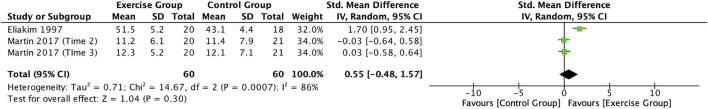
Forest plot of the effect of exercise on BALP in adolescents.

This study included two articles (Eliakim et al., 1997; Martin et al., 2017) describing the effect of exercise on BALP in adolescents. Meta-analysis showed a high degree of heterogeneity between the results of the included studies in the exercise group compared to the control group (*I* ^*2*^ = 86%, *p* = 0.0007), so the analysis was performed using a random-effects model (Figure 8), which showed a trend towards an increase in the level of BALP in the exercise intervention group compared to the control group (*SMD* = 0.55, 95% *CI*: -0.48–1.57, *p* = 0.30), but not significant (Figure 8).

#### 3.4.4 Effects of exercise on P1NP in adolescents

**FIGURE 9:**
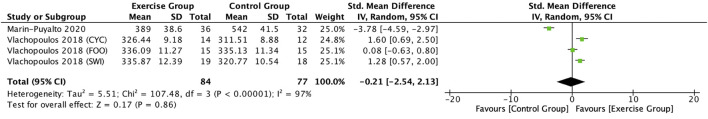
Forest plot of the effect of exercise on P1NP in adolescents. SWI, swimmers; FOO, footballers, CYC, cyclists.

This study included two articles (Vlachopoulos et al., 2018; Marin-Puyalto et al., 2020) describing the effect of exercise on P1NP in adolescents. Meta-analysis showed a high degree of heterogeneity between the results of the included studies in the exercise group compared to the control group (*I* ^*2*^ = 97%, *p* < 0.00001), so the analysis was performed using a random-effects model (Figure 9), and the results showed a trend toward a decrease in the level of P1NP in the exercise intervention group compared to the control group (*SMD* = −0.21, 95% *CI*: -2.54–2.13, *p* = 0.86), but not significant (Figure 9).

#### 3.4.5 Effects of exercise on OC in adolescents

**FIGURE 10:**
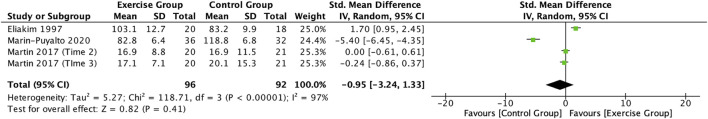
Forest plot of the effect of exercise on OC in adolescents.

Three articles (Eliakim et al., 1997; Martin et al., 2017; Marin-Puyalto et al., 2020) describing the effects of exercise on OC in adolescents were included in this study. Meta-analysis showed a high degree of heterogeneity among the results of the included studies (*I* ^*2*^ = 97%, *p* < 0.00001), so the analysis was conducted using a random-effects model (Figure 10), which showed a trend towards a decrease in OC levels in the exercise intervention group compared to the control group (*SMD* = −0.95, 95% *CI*: -3.24–1.33, *p* = 0.41), but it was not significant (Figure 10).

#### 3.4.6 Effects of exercise on CTX in adolescents

**FIGURE 11:**
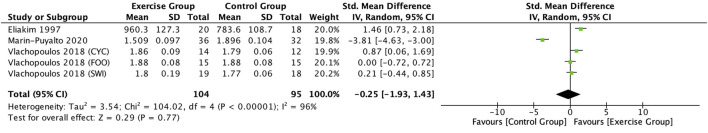
Forest plot of the effect of exercise on CTX in adolescents.

Three articles (Eliakim et al., 1997; Vlachopoulos et al., 2018; Marin-Puyalto et al., 2020) included in this study described the effect of exercise on CTX in adolescents. Meta-analysis showed a high degree of heterogeneity between the results of the included studies (*I* ^*2*^ = 96%, *p* < 0.00001), so the analysis was performed using a random-effects model (Figure 11), and the results showed a trend toward a decrease in the CTX levels in the exercise intervention group compared to the control group (*SMD* = −0.25, 95% *CI*: -1.93–1.43, *p* = 0.77), but not significant (Figure 11).

## 4 Discussion

The key to enhancing bone health is to engage in bone movement, an activity that is particularly important in promoting significant BMC accumulation during childhood and adolescence (Pasqualini et al., 2019). Study concludes that biological maturity is an important factor influencing youth exercise for BMD (Metcalf et al., 2020). There are also findings that aerobic plus resistance training improves bone metabolism in adolescents (Campos et al., 2014). However, the conclusions of the existing literature on the effects of exercise on BMC, BMD and bone metabolism markers in adolescent remain controversial due to differences in research paradigms. In this study, we conducted a meta-analysis of relevant RCTs published to date. RCTs are the gold standard for evaluating interventions and reside at the top of the evidence hierarchy for individual studies. The overall quality of the 15 RCTs included in the study was high, thus enhancing the reliability of the findings.

BMC often refers to the bone mineral content of a specific site or the whole body and is measured in g. BMD is usually measured in g/cm^2^, which is an important indicator of bone strength, reflecting the degree of calcium salt deposition in the bones. The present study revealed the different effects of exercise in promoting bone health in adolescents by comparing the effects of various exercise interventions on BMC and BMD at various sites. Overall, exercise had a significant effect on the improvement of overall BMC and BMD indices in adolescents in this study. Studies have shown that exercise actively promotes the activity and function of osteoblasts (Pasqualini et al., 2019; Smith, 2020), which in turn increases bone mass and enhances the efficiency of bone conversion, as well as triggering the release of sex hormones and insulin-like growth factor-1 and other substances (Aikawa et al., 2019), which promotes the accumulation of bone mass and the improvement of BMD.

Physical activity during skeletal development increases lumbar spine BMD, whereas more recent physical activity helps to maintain femoral neck BMD, and physical activity has different effects on BMD in different areas and at different ages, which are related to the processes of bone building and bone aging occurring at that time (Mello et al., 2022). A review of the literature suggests that regions with more bone trabeculae (lumbar spine) usually reflect exercise better (Petit et al., 2002), and there is also evidence that the increase in BMD with different exercises is found in regions with more cortical bone (Vuillemin et al., 2001). In this study, the exercise intervention group demonstrated significant positive effects on both lumbar spine and femoral neck BMC and BMD in adolescents, whereas there were no statistically significant effects on whole-body BMC and BMD. This is consistent with previous findings on the effects of exercise on BMD and BMC in postmenopausal women and children adolescents (Kelley et al., 2012; Wang Y. et al., 2023). Another study showed that physical activity had little effect on whole body BMD in overweight or obese subjects (Zouhal et al., 2021). This finding suggests that site-specific exercise interventions may act more directly on bone health in that region, and that differences in this effect are also related to the regional nature of the skeletal response to exercise mechanics (Petit et al., 2002; Okubo et al., 2017; Wang Y. et al., 2023). In the included studies, the experimenters intervened primarily through lower extremity-based exercise modalities (walking-running, high-intensity intervals, and jumping), which produce significant impact forces primarily on the hip and spine. Compared to other joints throughout the body, these areas of direct mechanical stress tend to show a more significant bone mass accumulation effect (Benedetti et al., 2018). The completion of athletic activities and technical movements is the result of the coordinated movement of bones and muscles in various parts of the body. During exercise, different skeletal muscle groups will be activated to different degrees according to the need, including the prime mover, antagonist, fixation and neutralization muscles, each of which produces unique biomechanical effects, which together promote the smooth progress of the movement (Cscs and Pt, 2006), and the various mechanical effects (tensile, shear, and extrusion forces) generated between the body and the ground, between the muscles, and between the skeleton and the muscles in the movement constitute an effective stimulus to the skeleton, which induces the generation of mechanical stresses. The generation of mechanical stress is induced. This mechanical stress stimulation in a moderate range can positively promote bone metabolism processes, triggering benign changes that enhance BMC and BMD (Wuyts et al., 2001), which in turn have an effect on the prevention and treatment of osteoporosis in adulthood (Ravi et al., 2020).

In the RCTs included in this study, a low-volume jump rope training session significantly increased femoral neck BMC compared with the control group (Arnett and Lutz, 2002); one core stability training session and one AquaPlyo session significantly increased lumbar spine and femoral neck BMD compared to the control group (Elnaggar et al., 2021; Elnaggar and Elfakharany, 2023). Different forms of exercise rely on different modes of organismal activity and produce different levels of stimulation in the organism (Berro et al., 2024). Witzke and others showed that sustained longer periods of augmented jump training increased peak bone mass during adolescent growth (Witzke and Snow, 2000). A study by Touban (Touban et al., 2022) confirmed that the cross-sectional area of the lumbar and abdominal core muscles is positively correlated with BMD, and that increased muscle mass and strength of the exercise also increases bone mass and its stiffness. This effect is valuable as it may prevent, delay or reverse bone loss. Benedetti (Benedetti et al., 2018) and others reported that resistance training with or without load may help to improve BMC and BMD in specific stimulated body regions. In addition, based on the properties of the piezoelectric effect, high volume and high-intensity strength training triggers a biochemical trigger in the skeleton due to the deformation or tension of the muscle contraction. signals. These biochemical signals stimulate cellular activity, leading to stress and calcium deposition at the training site (Cadore EL and Kruel, 2005). The remarkable effects of AquaPlyo training are also associated with its low-impact characteristics, reducing the risk of sports injuries while providing sufficient resistance to promote bone growth (Elnaggar and Elfakharany, 2023).

In the RCTs included in this study, aerobic exercise, resistance exercise, combination exercise, and whole-body vibration training, although they were able to elevate BMC and BMD in adolescents to some extent, the differences did not reach significance levels when compared to the control group. This is because although these exercises are beneficial for bone health, their individual effects may be mild and require longer or more intense interventions to show significant effects. One study investigated the effects of high-acceleration, maximal strength training on BMD in young adult women, completing 12 weeks of maximal strength training in deep squats, executed at 85%–90% of the maximal strength for 1 maximal repetition, with an emphasis on progressive loading and high acceleration in the centripetal phase (Mosti et al., 2014). The results of this study showed a significant increase in both lumbar spine and total hip BMD compared to the control group (Mosti et al., 2014). Another study reported that the increase in femoral neck and lumbar spine BMD was related to intensity compliance with resistance exercise, and that there should be greater loading with each repetition and adherence to a prolonged period of time in order to provide greater benefits to femoral neck and lumbar spine BMD (Gusi et al., 2006; Wang Z. et al., 2023). In this study, by incorporating the effects of a whole-body vibration training on skeletal health in adolescent athletes (Marin-Puyalto et al., 2020), although whole-body vibration training did not have a significant effect on BMD and BMC enhancement in a population of adolescent athletes when compared to a control group that performed their original exercise, a specific study noted that whole-body vibration training for a period of 20 weeks was able to significantly elevate whole body BMC in adolescents with and without Down’s syndrome levels (Villarroya et al., 2013), which suggests that this training modality has a positive effect on the improvement of bone mass under specific conditions or when targeting a specific population. In addition, studies with another population, postmenopausal women, have shown that whole-body vibration training produced significant positive effects on BMD in their lumbar spine and femoral neck regions (Von Stengel et al., 2011; Marín-Cascales et al., 2018). This further demonstrates the potential and application of whole-body vibration training as a non-pharmacological intervention in promoting bone health and preventing or alleviating osteoporosis, especially in populations with more significant effects on specific skeletal sites or specific physiological stages. High-intensity interval training failed to enhance BMD in adolescents, but instead showed a decreasing trend, which may be related to the specific parameters (intensity, duration, frequency) of the study design.

According to the guidelines, changes in bone turnover markers precede BMC and BMD, which should be measured 1 year after the start of treatment (Ward et al., 2017). However, in the studies we included, ten RCTs (Nichols et al., 2001; Arnett and Lutz, 2002; Kontulainen et al., 2002; Nogueira et al., 2014; Zribi et al., 2014; Marin-Puyalto et al., 2020; Elnaggar et al., 2021; Lau et al., 2021; Julian et al., 2022; Elnaggar and Elfakharany, 2023) measured BMC and BMD in less than 1 year, and even three studies (Elnaggar et al., 2021; Julian et al., 2022; Elnaggar and Elfakharany, 2023) measured them within 3 months. However, in this study we failed to find a significant difference in the improvement of bone metabolism markers (e.g., BALP, OC, P1AP, and CTX) in adolescents compared to controls, which may indicate that exercise has little effect on bone turnover markers in adolescents. This finding seems to be similar to the results of the Yan et al. (Yan et al., 2021) study, but at the same time we have seen studies that have reported that exercise contributes to bone turnover markers (Armamento-Villareal et al., 2020). Some bone formation markers, such as alkaline phosphatase (ALP), were not reported in the original study due to the limitations of the outcome metrics of the original study and therefore could not be analyzed. Moreover, there are fewer RCTs on the effects of exercise on bone metabolism in adolescents, and the results of this study are still not convincing enough overall.

The present study combined multiple forms of exercise intervention to analyze and compare BMC and BMD at different sites in adolescents without unfolding subgroup analyses of these forms of intervention because the refinement of the sample size categorization resulted in a smaller amount of combined effect sizes, which may have had an impact on the results. A meta-analysis has been performed to show that different forms of exercise intervention have different effects on BMD in middle-aged and older adults (Zhang S. et al., 2022). It has also been shown that the effect of different exercise interventions on skeletal metrics in adults and older adults varies according to the intensity of the intervention performed and the frequency of exercise (Kast et al., 2022; Zitzmann et al., 2022). RCTs on the effects of exercise on BMC, BMD, and bone metabolism in adolescents remain scarce, and future studies should be devoted to high-quality relevant RCTs leading to high-quality meta-analyses and further refinement of the analyses of intervention modality, period, frequency, duration, and intensity to address the dispersion of sample sizes while maintaining reasonable sample sizes, in order to comprehensively assess the effects of different intervention parameters on the adolescent body’s various BMC and BMD in different parts of the body, and the specific effects of exercise on various indices of bone metabolism, so as to provide more accurate and comprehensive health guidance and treatment strategies.

Limitations: (i) As the study analyzed BMC and BMD on different parts of the body, the refinement of the sample size classification may result in a smaller amount of combined effects, which may have a certain impact on the results, and therefore did not analyze the mode of intervention, the intervention period, the frequency of intervention, the duration of intervention, and the intensity of intervention; (ii) There are differences in the exercise intervention protocols of the included studies, which may have an impact when conducting subgroup analyses; (iii) The included Limited participants in the study, especially the study of exercise on bone metabolism in adolescents is less, the lack of big data support, the intervention effect of sports exercise on adolescents still need more related research.

## 5 Conclusion

This study was a systematic review and meta-analysis to assess the effects of an exercise intervention on BMC, bone mineral density BMD and bone metabolism in adolescents. Through a comprehensive analysis of 15 RCTs, the study covered 723 adolescents aged 10–19 years. The results showed that the exercise intervention had a significant effect in improving overall BMC and BMD in adolescents compared to the control group, particularly in the lumbar spine and femoral neck regions. However, the effects on total body bone mass and bone metabolism were not significant. These findings suggest that moderate exercise has a positive impact on adolescent bone health, especially during the critical period of growth and development, and helps to increase bone mass and improve bone metabolism, thereby reducing the risk of osteoporosis in adulthood.

Although the findings support the positive effects of exercise on adolescent bone health, these conclusions still need to be validated by more high-quality empirical studies due to the limitations of the number and quality of included studies. Future studies should further explore the specific effects of different exercise types, intensities, frequencies, and durations on adolescent bone health in order to provide more precise and comprehensive health guidance and intervention strategies for adolescents.

## Data Availability

The original contributions presented in the study are included in the article/supplementary material, further inquiries can be directed to the corresponding author.
